# Prefusion-stabilized SARS-CoV-2 spike reshapes antigenic hierarchy and antibody targeting against conserved and occluded epitopes

**DOI:** 10.1038/s41541-026-01464-2

**Published:** 2026-04-25

**Authors:** Shintaro Oishi, Ryutaro Kotaki, Hisham M. Dokainish, Saya Moriyama, Shinichiro Ota, Takayuki Matsumura, Tomohiro Takano, Taishi Onodera, Yu Adachi, Kazutaka Terahara, Masanori Isogawa, Kazuhiko Katayama, Takashi Sato, Masaharu Shinkai, Yoshimasa Takahashi

**Affiliations:** 1 Research Center for Vaccine Development, National Institute of Infectious Diseases, Japan Institute for Health Security, Tokyo, Japan; 2 Laboratory of Viral Infection Control, Department of Infection Control and Immunology, Ōmura Satoshi Memorial Institute & Graduate School of Infection Control Sciences, Kitasato University, Tokyo, Japan; 3 Department of Molecular Systems Immunology, University of Tokyo Pandemic Preparedness, Infection and Advanced Research Center (UTOPIA), Tokyo, Japan; 4 Respiratory Disease Center, Tokyo Shinagawa Hospital, Tokyo, Japan; 5 Department of Virology II, National Institute of Infectious Diseases, Japan Institute for Health Security, Tokyo, Japan; 6 Institute for Aqua Regeneration, Shinshu University, Nagano, Japan

**Keywords:** Computational biology and bioinformatics, Immunology, Microbiology

## Abstract

Receptor-binding domain (RBD) of the severe acute respiratory syndrome coronavirus 2 (SARS-CoV-2) spike contains multiple classes of antibody epitopes that are associated with diverse neutralizing activities. Although both natural infection and vaccination robustly elicit RBD-reactive and neutralizing antibodies, the spike antigenic structures presented to the immune system may differ, leading to qualitative differences in the antibody responses. Using large and well-controlled cohorts, we show that the neutralizing potency index (NPI), calculated as the ratio of neutralizing titer to RBD IgG titer, is approximately fivefold lower in vaccine recipients than in convalescent individuals, independent of disease severity, comorbidities, or demographic factors. This reduction in NPI is associated with enhanced antibody targeting to non-neutralizing, yet conserved and structurally occluded RBD epitope. Molecular dynamics (MD) simulations together with the binding assay demonstrate that the occluded epitope is allosterically exposed by stabilizing mutations introduced into the vaccine spike antigen, a process mediated by a highly extended RBD-up conformation. Collectively, our findings demonstrate that RBD conformational modulation by stabilizing mutations shapes vaccine antigenicity and likely alters the epitope landscape of antibody responses.

## Introduction

Neutralizing antibodies play a pivotal role in protection against viral infections^1^. During the coronavirus disease 2019 (COVID-19) pandemic, numerous clinical studies have consistently supported this concept, demonstrating that virus-neutralizing antibody titers serve as immune correlates of protection against symptomatic COVID-19, at least prior to the major antigenic shifts through emergence of SARS-CoV-2 Omicron variants^2–5^. Among the multiple vaccine platforms, mRNA vaccines have made a substantial contribution to global pandemic control, along with more than 94% effectiveness against symptomatic COVID-19 caused by ancestral SARS-CoV-2 strains^2,3^. Two representative mRNA vaccines, BNT162b2 (Comirnaty) and mRNA-1273 (Spikevax), utilize lipid nanoparticle (LNP)-encapsulated spike mRNAs encoding spike proteins that harbor two proline mutations (S2P) to stabilize the prefusion conformation^6–9^. Following vaccination, these LNP-mRNAs are translated into S2P spike protein within host cells, which then triggers multiple arms of the immune responses.

The SARS-CoV-2 spike protein forms a homotrimer composed of three functional domains: the S1 N-terminal domain (NTD), the S1 receptor-binding domain (RBD), and the S2 domain^9,10^. The RBD contains the receptor-binding motif (RBM), which directly engages the cellular receptor angiotensin-converting enzyme 2 (ACE2), making it primary target of potently neutralizing antibodies^11–14^. Although viral particles are primarily covered with prefusion spike proteins prior to cell entry^15^, the spike is conformationally flexible and exhibits heterogeneous conformation. In particular, the RBD dynamically converts between two reversible conformations, commonly referred to as the “up” and “down” states, at the apex of each spike trimer^13^.

RBD epitopes have been classified into four major classes based on their spatial locations, among which class 1 and 2 epitopes considerably overlap with the RBM and are frequently targeted by potent neutralizing antibodies^16,17^. In contrast, class 3 and 4 epitopes are located either outside the RBM or at the sites partially overlapped with RBM. While there are potently neutralizing antibodies binding class 3 or class 4 epitopes that overlap with RBM (e.g., LY-CoV1404^18^ and ADG20^19^), RBM non-overlapping epitopes represent typical targets of non- or poorly neutralizing RBD antibodies^14^. Compared to class 1-3 epitopes, class 4 epitopes are located at the internal surface and hidden under the down configuration^10,20,21^. This internal location conformationally restricts antibody access to the class 4 epitope under the down configuration, allowing the access of neutralizing class 4 antibodies solely in up configuration^22^. Further RBD conformational changes are required for exposing the internal bottom sites, the targets for the non-neutralizing class 4 antibodies, such as CR3022^20^. Indeed, CR3022/RBD complex overlaid on full spike protein is sterically constrained and unknown conformational changes are suggested to be involved for the CR3022 binding to RBD^20^. Thus, the RBD conformational landscape beyond the up configuration remains enigmatic so far, but the information is crucial for understanding non-neutralizing class 4 antibody binding. Since S2P mutations in vaccine antigen allosterically modulate the RBD conformational landscape^23^, it is also important to address how the S2P mutations affects the functional outcomes of vaccine-elicited antibody responses in vivo. However, the antigenic and immunogenic consequences of such conformational plasticity remain to be fully defined, especially in the vaccinees.

In addition to quantitative measurements of RBD-reactive antibodies, functional assessments of antibody responses have been explored in several studies. For example, the neutralizing potency index (NPI), defined as the neutralizing antibody titer normalized to the binding antibody titer, has been evaluated in COVID-19 patients and vaccinees^24,25^. These analyses revealed that higher NPI values of early plasma antibodies, rather than absolute quantities of neutralizing or binding antibodies, were associated with improved clinical outcomes, underscoring the association of the NPI values and protection at least under certain situations^24^. Similar NPI analyses have been applied to mRNA vaccine recipients and compared with those of convalescent individuals^26,27^. Notably, the vaccinees elicited antibodies with lower NPI, despite larger quantity of RBD-reactive antibodies than infection, suggesting that mRNA vaccination somehow induces non-neutralizing antibodies at higher ratios. However, the mechanistic basis for this observation has not been addressed, and further analyses using larger sample sizes and uniform sampling time points between vaccination and infection are the key to address the issue.

Here, we compared the NPI of plasma polyclonal antibodies collected at comparable time points following mRNA vaccination or natural infection using large cohorts (vaccination, *n* = 561; infection, *n* = 23). Consistent with previous reports^26,27^, we confirmed a significantly lower NPI in vaccine recipients than in convalescent individuals, independent of disease severity, comorbidities, or demographic factors. To elucidate the underlying mechanism, we performed epitope profiling of RBD-reactive polyclonal antibodies from the vaccinated and convalescent individuals (*n* = 24 and 23, respectively) by applying high-throughput approach. We revealed an accelerated antibody targeting toward an occluded class 4 epitope in the vaccine recipients, accounting for the reduced neutralizing potency of vaccine-elicited RBD antibodies. Finally, MD simulations and binding assay indicated that the class 4 epitope is allosterically exposed in the S2P-stabilized spike conformation, with fully extended RBD. Collectively, these results demonstrate that alterations in the RBD conformational landscape induced by stabilizing S2P mutations influence the epitope landscape of elicited antibody responses.

## Results

### mRNA vaccination elicits more robust RBD antibodies, yet lower NPI antibodies than natural infection

**Fig. 1:**
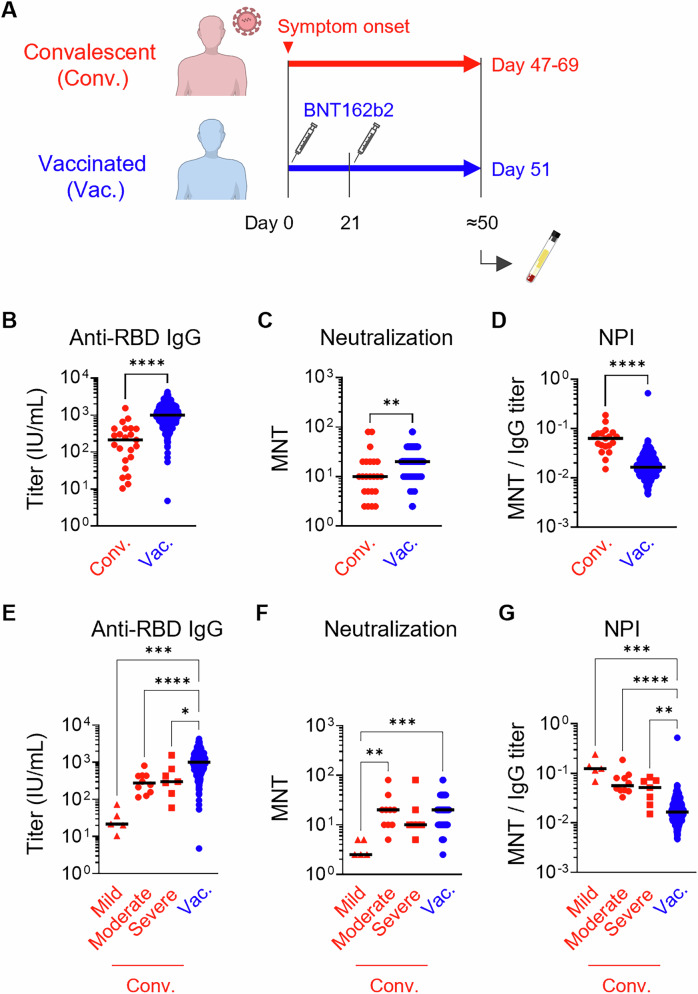
mRNA vaccination elicits robust RBD antibody responses with lower NPI than natural infection. **A** Schematic representation of the samples analyzed in this study. Both the convalescent and vaccinated groups were primed by the ancestral strain of SARS-CoV-2 spike as described in the main text. Plasma was collected at around 50 days after the first exposure to the antigen. Anti-RBD IgG titer (**B**), neutralization titer against authentic SARS-CoV-2 (**C**), and NPI calculated as the neutralization titer per the IgG titer (**D**) were compared between the convalescent and vaccinated plasma. Statistical analyses were performed with Mann–Whitney test (**p < 0.01, ****p < 0.0001). Anti-RBD IgG titer (**E**), neutralization titer against authentic SARS-CoV-2 (**F**), and NPI calculated as the neutralization titer per the IgG titer (**G**) were compared between the convalescent subdivided by symptomatic categories and vaccinated plasma. Statistical analyses were performed with Kruskal–Wallis test followed by Dunn’s multiple comparison test (*p < 0.05, **p < 0.01, ***p < 0.001, ****p < 0.0001). The dots represent data from each participant. The horizontal bars indicate median.

To compare antibody responses induced by infection and vaccination, we analyzed plasma samples from 23 convalescent individuals and 561 recipients of the ancestral BNT162b2 mRNA vaccine at approximately 50 days after their first exposure to SARS-CoV-2 antigens (Fig. 1A). For the vaccinated cohort, 68 of 629 participants were excluded due to suspected prior infection, as determined by anti-nucleoprotein titers (Fig. S1). The cut-off for anti-nucleoprotein titers was set 10-fold lower than commonly used thresholds to stringently exclude individuals with prior infection. Therefore, the vaccine recipients analyzed in this study were highly likely to have been primed solely by vaccination. Plasma samples from the COVID-19 patients (with mild, moderate, and severe symptoms) were collected at 47–69 days (median: 56 days) after their symptom onset. For the vaccinated group who received primary two doses of the vaccine with a 21-days-interval, plasma samples were collected uniformly at 51 days after the first dose of BNT162b2. The large sample size and comparable time points between vaccination and infection groups increase the sensitivity to assess the impacts of antigenic conformational changes in the vaccine antigens. The convalescent individuals were infected with the ancestral virus, as symptom onset preceded the emergence of the Alpha variant strain in Japan.

First, we compared RBD IgG titers and neutralizing antibody titers, measured with electrochemiluminescence immunoassay and authentic viral neutralization assay, respectively. Both titers were significantly higher in vaccine recipients than in convalescent individuals (Fig. 1B, C), indicating that mRNA vaccination induces more robust humoral immune responses in quantitative parameters. These robust antibody responses would contribute to high efficacy of mRNA vaccines. To assess qualitative differences in antibody responses, we calculated the NPI, defined as the neutralizing titer normalized to the anti-RBD IgG titer^24^. Despite the higher absolute antibody titers, vaccine recipients exhibited 5-fold lower NPI values than convalescent individuals (Fig. 1D), the findings nicely confirming the previous studies^26,27^.

A negative correlation between NPI and disease severity has been noted in COVID-19 patients^24^. We therefore subdivided convalescent individuals according to symptom severity and reanalyzed the links to antibody responses (Fig. 1E–G). Notably, NPI values in vaccine recipients were even lower than those observed in convalescent individuals with moderate or severe disease, who exhibited relatively low NPI among the convalescent group (Fig. 1G). Thus, mRNA vaccination induced robust antibody responses in quantity, but with comparatively lower NPI.

### The qualitative difference between vaccine- and infection-elicited antibodies is independent of demography and antibody avidity

**Fig. 2:**
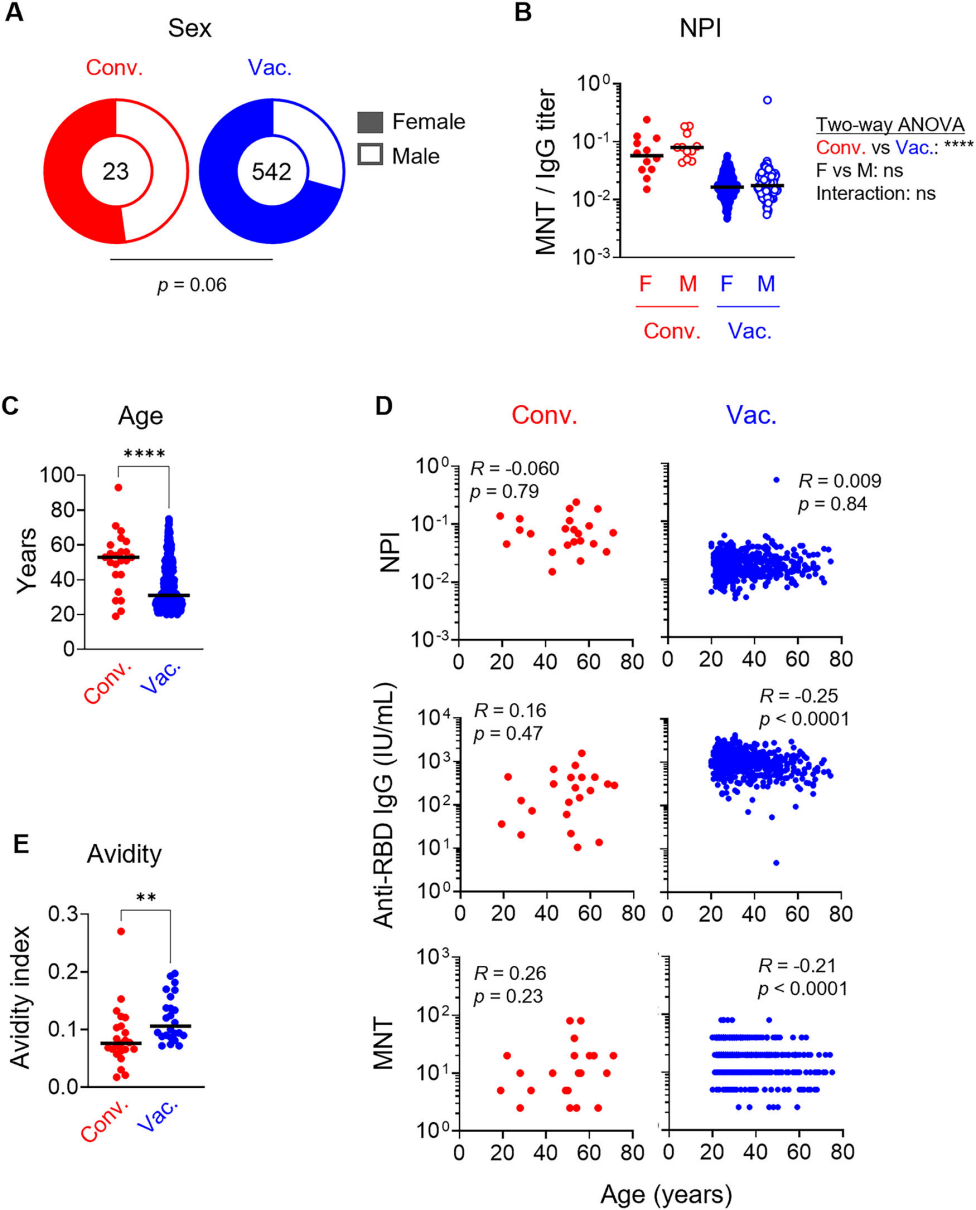
Demographic differences and avidity are not responsive for the lower NPI of the vaccinee plasma antibodies. **A** Female and male proportions of the vaccinated and convalescent groups are indicated. The numbers at the center indicate the numbers of the participants who consented to provide the information of their sex. Statistical analysis was performed with chai-squared test. **B** NPI of the vaccinated and convalescent groups analyzed in Fig. 1D were further subdivided into female and male. Statistical analyses were performed with two-way ANOVA (****p < 0.0001). **C** Distributions of ages in the convalescent and vaccinated groups are described. Statistical analysis was performed with Mann–Whitney test (****p < 0.0001). **D** Correlation of age and NPI are analyzed in the convalescent and vaccinated groups. Statistical analyses were performed with Spearman’s test. **E** Avidity of plasma antibodies against the ancestral RBD in the convalescent and vaccinated groups were measured with ELISA. The dots represent data from each participant. The horizontal bars indicate median.

To identify factors contributing to the difference in NPI between vaccinated and convalescent individuals, we first examined potential demographic effects. Although the vaccinated cohort included a higher proportion of female participants than the convalescent group (Fig. 2A), NPI values were comparable between males and females within each group (Fig. 2B). The two cohorts also differed in age distribution (Fig. 2C). Anti-RBD IgG and neutralizing antibody titers negatively correlated with age in the vaccinated cohort (Fig. 2D); however, age did not correlate with NPI. In the convalescent cohort, neither antibody titers nor NPI correlated with age, possibly due to the limited sample size. These results indicated that demographic differences did not account for the observed NPI reduction in the vaccinees.

Because the antibody-neutralizing activity is influenced by both binding avidity and epitope specificity, we first assessed the avidity of plasma antibodies. For direct comparison, we selected a representative subset of vaccinated samples in the way to preserve the distributions of RBD IgG and neutralizing titers. These selected samples showed no bias in antibody titers or NPI relative to the original vaccinated cohort, while retaining significant differences from the convalescent group (Fig. S2). Using the selected samples, we compared the avidity of plasma RBD IgG antibodies between vaccinated and convalescent individuals. We measured avidity index as fold change of RBD-binding IgG titers in the presence of disruptive urea from those without urea. Vaccination induced significantly higher antibody avidity than infection (Fig. 2E), despite the lower NPI observed in vaccine recipients. These findings indicate that antibody avidity does not explain the reduced neutralizing potency per binding antibody after vaccination.

### Vaccine-induced antibodies increasingly bind to conserved class 4 RBD epitopes

**Fig. 3:**
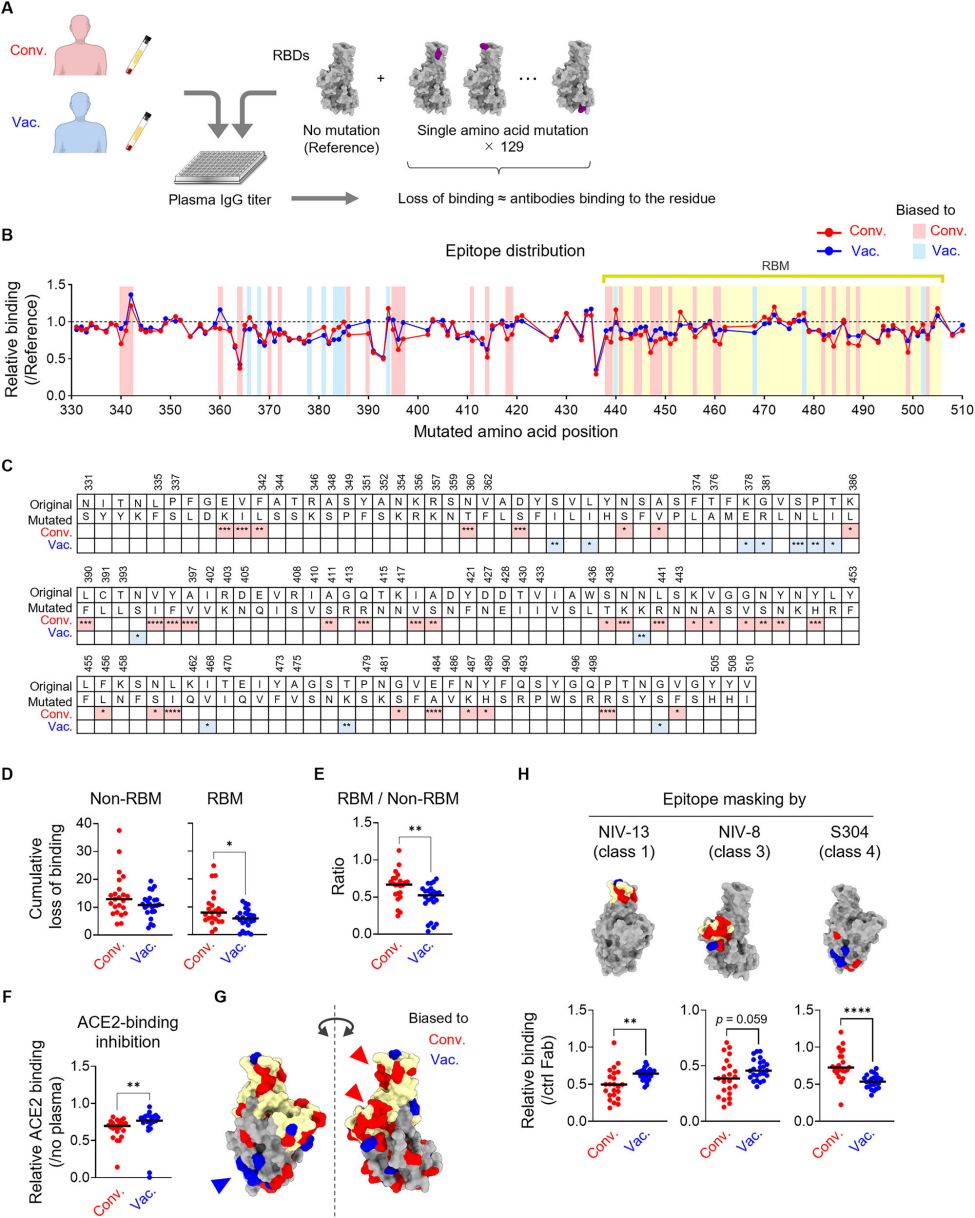
Vaccine-induced antibodies are preferentially directed toward class 4 RBD epitopes compared with infection. **A** Schema of epitope mapping using single mutation RBD panel is described. **B** Relative binding to each single mutation, normalized by binding to the ancestral RBD, was analyzed for the convalescent and vaccinated plasma. The amino acid residues where relative binding in convalescent plasma was significantly lower than in vaccinated plasma are highlighted in red, indicating epitopes preferentially targeted by convalescent plasma compared with vaccinated plasma. Conversely, the residues highlighted in blue indicate epitopes preferentially targeted by the vaccinees. RBM is highlighted in yellow. The dots indicate median values of each group. **C** Details of single mutation RBD panel assay is described. Position of mutation, amino acid substitutions, and significant difference in binding between the convalescent and vaccinated plasma are described. **D** Cumulative loss of binding throughout non-RBM residues and RBM residues was compared between the convalescent and vaccinated groups. **E** Ratio of the cumulative loss of binding analyzed in **D** was calculated. **F** Competitive binding inhibition of plasma antibodies against human ACE2 was assessed. Plasma antibodies were diluted to the equivalent anti-RBD IgG titers to compare proportion of RBM-binding antibodies. **G** RBD residues preferentially targeted by antibodies in convalescent (red) or vaccinated (blue) individuals, as highlighted in **B** and **C**, were located on 3D structure. RBM is highlighted in yellow. Arrowheads indicate clusters of the preferentially targeted residues by the convalescent (red) or vaccinated (blue) plasma antibodies. **H** Epitope competition of plasma antibodies with Fab from indicated monoclonal antibodies were analyzed. Amino acid residues highlighted in **G** overlapping with epitopes of the Fab are shown in upper images. These epitopes on RBD were masked by each Fab prior to plasma antibody binding. Binding of plasma antibodies were normalized to that competed by non-binding control Fab. Statistical analyses were performed with Mann–Whitney test (*p < 0.05, **p < 0.01, ***p < 0.001, ****p < 0.0001). The dots and the horizontal bars in **D**–**H** represent data from each participant and median, respectively.

We next investigated whether any shifts in the epitope landscape contribute to the different NPI in antibody responses. To comprehensively profile RBD epitopes, we generated a panel of 129 RBD variants based on the ancestral strain, each containing a single amino acid substitution which was most prevalent at each position according to the GISAID database as of February 2022. We tested binding profiles of monoclonal antibodies, REGN10933 and COVA1-16, to confirm that all the RBDs assembled properly. Each mutant RBD showed binding by either or both antibodies at the same level as the ancestral RBD (Fig. S3). It should be noted that COVA1-16 lost binding by mutations which are not its epitope, such as D364S, S366I, C391L, and T393L. These mutations may affect local conformation around the mutated residue but not entire RBD structure, because REGN10933 still bound normally to these mutants. Polyclonal IgG binding to each mutated RBD was quantified using a multiplex bead-based assay and normalized to binding against the ancestral RBD to assess loss of binding by the mutations at each residue (Fig. 3A). The degree of binding loss reflects the relative abundance of antibodies targeting the mutated residue in polyclonal antibodies.

Comparison of epitope profiles revealed distinct biases on the epitope preference between convalescent and vaccinated antibodies (Fig. 3B, C). Because neutralizing antibodies predominantly target RBM, we compared cumulative binding loss at RBM versus non-RBM residues. Vaccine antibodies exhibited significantly lower cumulative binding loss at RBM residues (Fig. 3D) and a reduced RBM-to–non-RBM binding ratio compared with convalescent antibodies (Fig. 3E), indicating a preferential targeting of non-RBM regions following vaccination. To functionally validate this observation, we assessed the ability of plasma antibodies to inhibit ACE2–RBD interactions under the conditions of comparable RBD IgG titers, specifically, all plasma samples were diluted to the same RBD IgG titer prior to the experiment. Consistent with the epitope profiling data, plasma antibodies from vaccine recipients showed less ACE2-binding inhibitory activities compared with convalescent plasma (Fig. 3F). These results clearly confirm that vaccine-induced antibodies are more frequently directed toward non-RBM regions of the RBD, nicely accounting for the lower NPI.

To clarify detailed epitopes differently targeted by the convalescent and vaccinated groups, we further analyzed distributions of residues that are significantly biased in either group on a 3D structure of the RBD (Fig. 3G). Some of the biased residues, which were distributed sparsely in a linear sequence (Fig. 3C), were clustered on the structure as indicated in Fig. 3G with arrowheads. Two clustered residues biased in the convalescent group were on the RBM, whereas those biased in the vaccinated group were outside RBM. The convalescent clusters were largely overlapped with class 1 and class 3 epitopes, where neutralizing antibodies bind. The residues biased in the vaccine group belong to class 4 epitope, typically bound by non-neutralizing antibodies. Hence, we further performed competitive binding assay using class 1 (NIV-13)^28^, class 3 (NIV-8)^28^, and class 4 (S304)^21,29^ monoclonal antibody clones as the competitors to assess whether plasma antibodies from the convalescent and vaccinated groups are indeed directed towards these epitopes. Consistent with the results from RBD mutant assay, the antibody binding in the convalescent group was more strongly competed by the class 1 antibody than the vaccinated group (Fig. 3H). Likewise, the vaccine antibodies were more strongly competed by the class 4 antibody than the convalescent antibodies. Together, these epitope mapping data demonstrate that vaccine-elicited antibodies are more directed to non-neutralizing class 4 epitopes, nicely accounting for the reduced NPI values.

### Proline mutations in spike antigens enhance antibody accessibility to occluded class 4 epitopes

The observed differences in epitope specificity suggest that RBD epitopes are differentially exposed on spike antigens during infection and vaccination. Because the RBD dynamically adopts up and down conformations on the spike trimer, we mapped convalescent- and vaccine-biased epitopes onto spike structures containing either up- or down-RBD conformations (Fig. S4A). Class 1 and class 3 epitopes enriched in convalescent antibodies were accessible in both RBD conformations (Fig. S4B). In contrast, the vaccine-biased class 4 epitopes remained largely occluded in both up and down states, indicating that simple RBD up/down transitions do not account for the enhanced targeting of class 4 epitopes in vaccine recipients. Consistent with this interpretation, previous studies have shown that additional conformational rearrangements beyond the canonical up/down states are required for the access by non-neutralizing class 4 antibody, represented by CR3022 clone^20^.

**Fig. 4:**
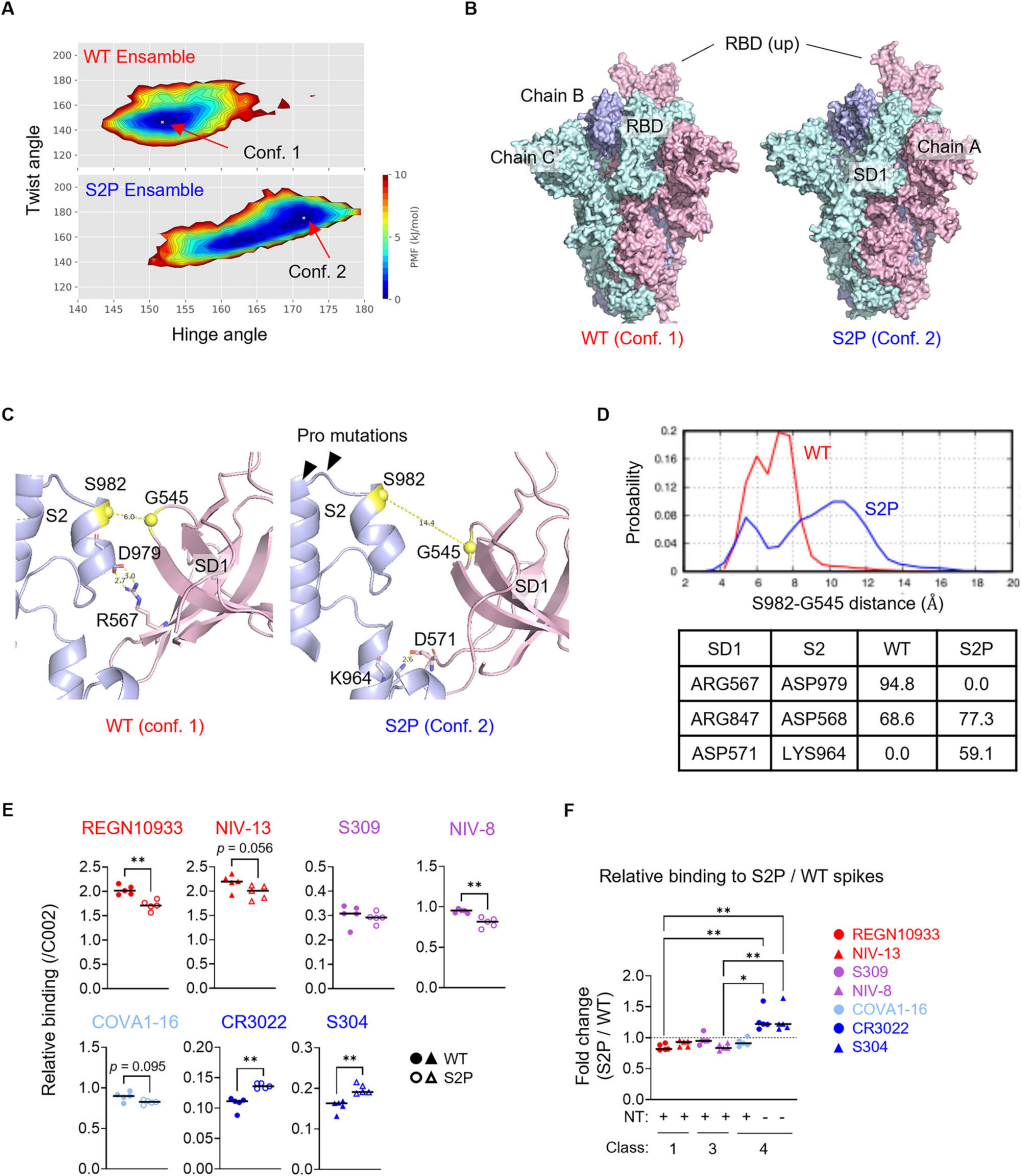
S2P mutations enhance conformational changes in spike to expose class 4 epitopes on RBD. **A** Free-energy landscapes projected onto the RBD–SD1 hinge angle (degree of opening) and RBD twist angle (rotational displacement) for WT (top) and S2P (bottom) spike simulations, all initiated from an Up conformation. The S2P mutation shifts the dominant ensemble toward larger hinge and twist angles, corresponding to more open Up conformations. White crosses denote representative dominant free-energy basins structures. **B** Representative spike trimer structures extracted from the dominant Up basins of WT and S2P simulations, highlighting increased RBD opening in the S2P ensemble. **C** Representative conformations illustrating altered SD1–S2 interactions in WT and S2P, including key salt bridges and hydrogen bonds. **D** Probability distributions of the SD1–S2 (G545–S982) Cα distance across 500 ns simulations, showing a broader and shifted distribution in S2P, consistent with weakened inter-domain coupling. The accompanying table summarizes the occupancy of key SD1–S2 interactions (HB and salt bridge) across the ensemble. **E** Binding titer of monoclonal antibodies against spike with or without S2P mutation were normalized to those of C002 antibody. Statistical analyses were performed with Mann–Whitney test (**p < 0.01). **F** Fold change of the binding to S2P over non-S2P spike are plotted for each antibody. Statistical analyses were performed with Kruskal–Wallis test followed by Dunn’s multiple comparison test (*p < 0.05, **p < 0.01). The dots and horizontal bars represent data from five independent experiments and median, respectively.

The spike mRNA sequence used in the vaccine contains the S2P mutations that stabilize the prefusion conformation^6–9^. Although these mutations prevent the transition to the postfusion state, their broader effects on spike conformational dynamics remain incompletely characterized. To assess how the prefusion-stabilizing S2P mutations affect spike conformational dynamics, we performed fully glycosylated all-atom MD simulations of the prefusion 1-up spike trimer (Fig. S5A), initiating all trajectories from an RBD-up conformation. Because all simulations began from the same up geometry, our analysis focused on redistribution within the up-state ensemble, rather than transitions between up and down states. Projection of the trajectories onto the RBD–SD1 hinge (RBD opening) and twist (RBD rotation) angles (Fig. S5B) revealed a clear reshaping of the conformational free-energy landscape upon introduction of the S2P mutations (Fig. 4A). While the wild-type (WT) ensemble remained confined to a relatively compact region corresponding to moderately open up conformations, the S2P ensemble distributed in a broader range of hinge and twist angles. Importantly, the dominant basin in the S2P landscape was shifted toward more open conformations, rather than representing a newly formed structural state. Representative structures extracted from the dominant basins illustrated this redistribution at the structural level (Fig. 4B). Compared with WT, the S2P ensemble exhibited increased separation and reorientation of the up RBD relative to the central trimer axis, consistent with enhanced opening within the up-state ensemble.

Analysis of hinge and twist angle distributions across all three RBDs showed that the down protomers remained comparatively constrained, while the up RBD exhibited expanded sampling in the S2P simulations (Fig. S5C). Two-dimensional projections further showed minimal correlation between large-amplitude opening of the up RBD and hinge motions of the adjacent down RBD (Fig. S5D), accompanied by altered inter-RBD contacts (Fig. S6A,B). These findings indicate that the ensemble redistribution induced by the S2P mutations reflects a local mechanical effect on the up protomer, rather than cooperative opening or destabilization of the trimer.

To identify the structural origin of this ensemble redistribution, we examined interactions between subdomain 1 (SD1) and the S2 region, which have been implicated in constraining RBD motion. Structural comparison of representative WT and S2P conformations highlighted differences in key SD1–S2 contacts, including reduced persistence of stabilizing salt bridges (R567–D979) and hydrogen bonds in the S2P mutant (Fig. 4C, D). Consistent with these observations, the probability distribution of the SD1–S2 Cα distance showed a clear shift toward larger values and increased breadth in the S2P simulations relative to WT (Fig. 4D). This broadening indicates weakened mechanical coupling between SD1 and S2, thereby reducing constraints on RBD motion. We further analyzed interactions in the helix region surrounding the mutation sites, where disruption of intra-helix contacts involving K964_S968 (72% in WT vs. 22% in S2P) allows formation of an inter-domain salt bridge with SD1 (K964–D571; Fig. 4C, D). This creates a new anchoring interaction at the lower S2–SD1 interface, increasing mobility at the upper SD1–S2 interface and contributing to disruption of the R567–D979 salt bridge. Together, these results demonstrate that the nearby S2P mutation promotes enhanced up-state opening by locally loosening SD1–S2 interactions rather than by inducing a global structural rearrangement. Notably, HDX-MS data from Costello et al. indicate increased solvent accessibility at the S2 interface of the S2P spike^30^, consistent with our MD simulations showing enhanced motions and exposure in the Up chain, while Down chains remain more constrained.

Further, analysis of inter-domain interactions within S1 revealed a reduction in persistence of contacts between the up RBD and neighboring RBDs and NTDs in the S2P ensemble (Fig. S6C, D). Correspondingly, inter-RBD and inter-NTD center-of-mass distance distributions were broader in the S2P simulations, indicating increased conformational heterogeneity within S1 without disruption of the prefusion architecture, thereby facilitating RBD opening. The enhanced opening of up-RBDs on S2P spike antigens may have increased accessibility of class 4 antibodies during the vaccination.

To experimentally validate this prediction, we compared the binding of representative class 1–4 monoclonal antibodies to WT and S2P spike proteins. Binding of class 1 (REGN10933 and NIV-13)^28,31^, class 2 (C002)^16,32^, class 3 (S309 and NIV-8)^28,29^, and class 4 (COVA1-16, CR3022, and S304)^20,21,29,33,34^ antibodies to the spike proteins were assessed. Binding signals were normalized to those of the class 2 antibody C002 to directly compare binding to the spikes irrespective of post-fusion conformation frequency, which is potentially different with or without S2P. Two of the three class 4 antibodies, including CR3022 and S304, exhibited increased binding to the S2P-stabilized spike compared with the WT spike (Fig. 4E), resulting in higher fold changes in binding (Fig. 4F). In contrast, binding of another class 4 antibody, COVA1-16, was not enhanced by the S2P mutations. While CR3022 and S304 bind epitopes relatively far from RBM (Fig. S7) and have no or very weak neutralizing activity, COVA1-16 binds a non-RBM epitope proximal to the RBM and competes with ACE2 binding^20,21,33,34^. Thus, COVA1-16 may access its epitope even in the absence of the S2P mutations. In addition to the class 4 antibodies, class 3 antibodies, S309 and NIV-8, exhibited different kinetics: S309 comparably bound the WT and S2P spikes, while NIV-8 showed higher binding towards the WT spike (Fig. 4F). NIV-8 binds an epitope overlapping RBM, while S309 epitope is largely non-RBM (Fig. S7). These epitopes might be differently exposed between the WT and S2P spikes.

Together, these results demonstrate that the S2P mutations incorporated into vaccine spike antigens likely enhance exposure of occluded class 4 epitopes, thereby biasing antibody responses toward non-neutralizing regions of the RBD compared to infection-induced antibodies.

## Discussion

Vaccine immunogenicity is determined by the combinatory effects of two key components; antigens and platforms. The immunogenicity of LNP-mRNA vaccines was superior to that of natural infection and other vaccine platforms (e.g., viral vector and inactivated vaccines) even when the same spike protein was used as the vaccine antigen^35^. On the other hand, the same mRNA vaccine platforms encoding either spike or RBD protein showed the comparable immunogenicity to elicit antibody responses^7^. These immunogenic evidence in clinical studies demonstrate that vaccine platform, rather than the type of antigens, is a dominant parameter for the immunogenicity that controls the quantity of antibodies produced. Indeed, mouse studies clearly identified an ionized lipid, one out of four lipid components for LNP, on determining the mRNA vaccine immunogenicity^36,37^. The strong immunogenicity of the LNP-mRNA platform resulted in the robust elicitation of RBD-reactive and neutralizing antibody responses in the vaccinees and contributed to the pandemic control.

Nonetheless, the strong potency of SARS-CoV-2 mRNA vaccines would not be achieved without the use of S2P spike sequence that stabilizes the prefusion spike conformation and synergistically enhance the potency to elicit RBD-reactive and neutralizing antibody responses. Despite the quantitative improvement, previous studies observed relative defects in the neutralizing potency per the binding antibodies after mRNA vaccination compared to natural infection. Indeed, the same phenomenon was observed in this study using the larger sizes of cohort with the arranged time points in both vaccine recipients and convalescent individuals. Our high-throughput epitope profiling analysis further revealed a biased antibody targeting to non-neutralizing, yet conserved and occluded epitopes behind the process, compatible with a previous study which showed mRNA-1273 vaccination induces antibodies with broader epitope distributions than infection^38^. Moreover, we revealed that the distinct antibody targeting was due to the altered RBD conformation through prefusion stabilizing S2P mutations, the notion supported by both MD simulations and the binding assay.

Accelerated induction of non-neutralizing antibodies to conserved and occluded epitopes contributes to reduction of NPI values in the RBD-reactive antibodies. However, it is unlikely that the reduction of NPI proportionally attenuates protective function of antibodies. Non-neutralizing, RBD-reactive IgG antibodies can mediate protection through IgG Fc-mediated pathway, the best example derived from class 3 antibody, S309, which is the prototype antibody clone for sotrovimab, approved antibody therapeutics for COVID-19. Whereas the S309 exhibit relatively low neutralizing activity, it mediates strong protection through Fc-mediated pathway in animal models^39,40^. Despite the modest neutralizing activity, S309 exhibits potent protective efficacy even when the activity was compared to highly neutralizing antibodies, highlighting the contribution of non-neutralizing pathway on protection in vivo. Furthermore, S309 provides protection against SARS-CoV-2 Omicron variants in animal models through Fc-mediated pathways, despite S309 showing undetectable neutralizing potency against these variants^41^. Potency of Fc-mediated protective functions differs between antibodies probably depending on binding orientations and relative Fc positioning to Fc receptors on effector cells^29^. Some class 4 antibodies, S304^29^ and CR3022^42^, also have been reported to exhibit Fc-mediated functions. Fc-mediated protection of S309 against Omicron variants was also demonstrated in real world setting by reduced hospitalization and death during Omicron waves in patients treated with sotrovimab^43,44^. Given the conserved nature of class 4 epitopes among SARS-CoV-2 variants, the robust elicitation of class 4 antibodies by mRNA vaccines may increase the breadth of protection against SARS-CoV-2 variants through the Fc-mediated functions. The class 4 epitope was conserved probably because of mutational constrains for molecular stability of RBD^45^, besides less immunological pressure by non-neutralizing class 4 antibodies, which contribute to protection by blocking late stage of virus replication. Further studies will be needed to clarify potency of non-neutralizing class 4 antibodies for protection and their effects on viral evolution.

After the primary series of the ancestral mRNA vaccination, memory B cells cross-reactive to Omicron variants developed over time and contributed to Omicron-reactive antibody production following additional booster vaccination^46–49^. These cross-reactive B cells largely bind epitopes which is subdominant during the early responses after the primary vaccination, as represented by class 4 epitope^50^. The antibody epitopes can be greatly shifted toward subdominant ones by antibody feedback after additional ancestral booster, where pre-existing antibodies specific to dominant epitopes modulate subsequent B cell selection to favor subdominant ones^50,51^. The increased accessibility of antibodies to occluded epitopes in S2P spike antigens potentially increases the chance to select B cells against subdominant class 4 epitopes under the antibody feedback process, indirectly contributing to the expanded breath of antibody after additional booster towards Omicron strains.

Dynamic conformations of proteins are hardly characterized by experimental approaches because of their heterogenous and probabilistic nature. Instead, our simulations provide a molecular explanation for how the S2P mutations alter spike conformational behavior in ways that are directly relevant to antibody recognition. While previous cryo-EM and biochemical studies established that S2P preserves the prefusion architecture and increases the prevalence of RBD-up states in vaccine antigens, they have not resolved how flexible nor accessible the up state is once formed. Here, we showed that S2P does not create a new spike conformation but instead redistributes the existing up-state ensemble toward more open and rotationally permissive geometries. This effect originated from weakened interactions at the SD1–S2 interface, which normally constrain RBD motion. In the S2P spike, disruption of stabilizing salt bridges and hydrogen-bond networks locally loosened this coupling, allowing the up RBD to sample larger separations and orientations relative to the trimer core. Importantly, this enhanced mobility was largely confined to the up protomer and did not require cooperative opening of neighboring RBDs, consistent with the preserved global stability of the prefusion spike observed experimentally.

These conformational changes have clear implications for antibody accessibility. Structural studies of the class 4 antibody CR3022 and related antibodies have shown that productive binding requires RBD conformations that extend beyond a canonical up state, involving additional opening and rotation to avoid steric clashes with adjacent domains or even 2 up formation^20,52,53^. Although such fully accessible conformations were not explicitly reached on the present simulation timescale, the S2P mutations shifted the up-state ensemble toward geometries that more closely resemble these binding-competent states, increasing the likelihood of transient exposure of conserved, cryptic epitopes. Notably, using enhanced sampling approach, we have previously shown the formation of highly open up conformation in S2P^54^.

This mechanism provides a structural basis for the preferential elicitation of class 4 antibodies following vaccination with prefusion-stabilized spike, in contrast to natural infection. While down-RBD conformation of spike proteins buries RBM into the trimer interface, which may avoid neutralizing antibody responses^13^, we and others observed neutralizing antibodies in convalescent and vaccinated cohorts. This indicates that spike antigens in the infection and vaccination harbor up-RBDs. A part of class 4 epitopes is sterically constrained even in the up-RBD structure^20^. Our MD simulations suggested that rather than simply increasing the number of up RBDs, the S2P mutations alter how the up state is presented, favoring more open and dynamically accessible conformations during antigen exposure. Together with the experimental findings presented here, our results show that the immunological impact of S2P arises from a subtle reprogramming of spike conformational behavior that enhances epitope accessibility while preserving overall prefusion stability. This suggests the need for designing stabilizing immunogens that balance structural integrity with controlled conformational flexibility. In this context, mutations can be selected not only to maintain prefusion stability but also to modulate epitope presentation, enabling targeted exposure of conserved or neutralization-sensitive regions while limiting immunodominance of less protective sites.

Our study has several limitations. The MD simulations performed in this study were limited to a 500 ns timescale and started from an RBD-Up conformation, therefore did not capture rare global opening events nor directly model antibody binding. Nevertheless, the consistent differences observed between WT and S2P across multiple structural measures indicate that S2P alters spike conformational behavior in a way that aligns with experimental observations of class 4 antibody accessibility. Although our binding data consistently support the altered epitope landscape between WT and S2P spikes, we cannot exclude the possibility that other unknown factors are also involved in the difference in epitope profiles of antibodies between vaccination and infection, such as multiple aspects of difference between infected viruses and mRNA vaccines in the antigen presentation to immune systems under distinct inflammatory conditions. Direct comparison of antibody responses induced by WT and S2P spikes under identical platform is the key to address this point, e.g., adenovirus vector vaccines, ChAdOx1 (WT spike antigen) and Ad26.COV2.S (S2P spike antigen). Also, we only analyzed antibody responses induced by the ancestral strain antigen. In the current situation, we need to consider responses to Omicron variant strains which are currently circulating and used for vaccine strains. The mutations in variant spikes affect their structure and conformational dynamics^55,56^, thus effective strategy for controlling antigen conformation is likely different between the ancestral and Omicron variants.

## Methods

### Enrollment of human participants

COVID-19 convalescent and BNT162b2 vaccinated participants were enrolled as previously described at Tokyo Shinagawa Hospital^25,57^.

The present study has been approved by the Institutional Review Board of the National Institute of Infectious Diseases and the Institutional Review Board of Tokyo Shinagawa Hospital (Permit numbers: 1237, 1604, 20-A-08, and 20-A-33). All experiments were performed according to the Declaration of Helsinki. All participants provided written informed consent before enrollment.

### Blood collection

Blood samples were collected as described previously from COVID-19 convalescent patients and COVID-19 mRNA vaccinated health care workers^25,46,57^. Blood was drawn by Vacutainer CPT tubes (BD biosciences) followed by centrifugation at 1800×*g* for 20 min. Peripheral blood mononuclear cells were suspended in plasma and were transferred into conical tubes. After centrifugation at 300 × *g* for 15 min, plasma was harvested into another tube followed by further centrifugation at 800 × *g* for 15 min. The plasma was collected into another tube and were heat inactivated at 56 °C for 30 min.

### Electrochemiluminescence immunoassay

Anti-nucleoprotein antibody titers were measured with Cobas e411 plus (Roche) as previously described^46,58^.

Anti-RBD IgG titers were measured with mesoscale V-PLEX assay (Meso Scale Discovery) according to the manufacturer’s instruction as described previously^46^. The V-PLEX plates (SARS-COV-2 Panel 22) were blocked with Blocker A solution (Meso Scale Discovery) for 1 h. After the plates were washed with Wash buffer for three times, plasma samples and reference with known antibody titer diluted in Diluent 100 (Meso Scale Discovery) were added into the plates. The plates were incubated at room temperature for 2 h. The plates were washed with Wash buffer for three times. Sulfo-Tag Anti-human IgG (Meso Scale Discovery) diluted in Diluent 100 were added into the plates followed by incubation at room temperature for 1 hour. After the plates were washed with Wash buffer for three times, MSD Gold read buffer B (Meso Scale Discovery) was added into the plates. Electrochemiluminescence was immediately measured by MESO Quick Plex SQ 120 (Meso Scale Discovery).

Competitive inhibition of ACE2 binding to RBD by plasma antibodies was also measured with mesoscale V-PLEX kit (Meso Scale Discovery) as described previously^59^. V-PLEX plates (SARS-CoV-2 Panel 22) were blocked with the blocking buffer for 1 h followed by washing with the Wash buffer. The plates were incubated with plasma diluted in Diluent 100 for 1 h. Then, SULFO-TAG Human ACE2 Protein (Meso Scale Discovery) was added without washing, followed by incubation at room temperature for 1 h. The plates were washed with the Wash buffer, followed by the addition of MSD Gold read buffer B (Meso Scale Discovery). Electrochemiluminescence was immediately measured using MESO QuickPlex SQ120 (Meso Scale Discovery). ACE2-binding signals were normalized to those obtained in blank wells (only the diluent was added instead of diluted plasma).

### Neutralization assay

Plasma neutralization titers were measured against authentic SARS-CoV-2 virus (the ancestral WK-521 strain) as described previously^25,46^. Briefly, plasma samples were serially diluted in high-glucose Dulbecco’s modified Eagle’s medium (DMEM) supplemented with 2% fetal bovine serum (FBS) and penicillin/streptomycin (100 U/mL) in 96-well plates and were mixed with 100 TCID_50_ of authentic SARS-CoV-2 virus (hCoV-19/Japan/TY-WK-521/2020, ancestral strain). The plates were incubated at 37 °C for 1 h. The virus-plasma mixtures were placed on VeroE6/ TMPRSS2 cells (JCRB1819) seeded in another 96-well plates and cultured at 37°C with 5% CO_2_ for 5 days. After the culture, the cells were fixed with 20% formalin (Fujifilm Wako Pure Chemicals) and were stained with crystal violet solution (Sigma-Aldrich). The mean cutoff dilution index with >50% cytopathic effect from two to four multiplicate series was presented as the neutralizing titer.

### Avidity measurement by ELISA

Avidity of plasma IgG antibodies against SARS-CoV-2 spike RBD was measured by ELISA with urea-washing step as described previously^25^. Spike RBD proteins were coated on Nunc maxisorp plates (Thermo Fisher Scientific) at 4 °C overnight. The plates were washed with PBS-T for three times using Agilent plate washer (Agilent) followed by blocking with 1% BSA/PBS at room temperature for 1 h. After the blocking buffer was removed, serially diluted plasma samples in Diluent 100 (Meso Scale Discovery) were added into the plates. Two hours later, the plates were washed with PBS-T for three times followed by detachment of low avidity antibodies with 7 M urea/PBS or control PBS at room temperature for 1 h. After washing with PBS-T for three times, HRP-conjugated goat anti-human IgG antibody diluted in 1% BSA/PBS-T was added. One hour later, the plates were washed with PBS-T for three times. OPD substrate was added into the plates followed by stopping the enzyme reaction with 2 N HCl. Absorbance at 490 nm was measured by Epoch 2 Microplate Spectrophotometer (BioTek). Ratio of binding titer with or without the urea-treatment was calculated as avidity index. The binding titers were measured at a constant OD value for all samples to calculate avidity at the same RBD IgG titer among all samples.

### Epitope analysis by single mutation RBD panel

Binding profile of plasma antibodies was analyzed with Luminex multiplex assay using single mutation RBD panel.

Biotinylated RBDs with single amino acid mutation listed in Fig. 3C were prepared as previously described^28^. Briefly, pCAGGS plasmid vectors coding a signal peptide, RBD (amino acids from spike: 331-529), His-tag, and Avi-tag were co-transfected with BirA plasmid (Addgene) to Expi293F cells (Thermo Fisher Scientific) followed by culture in Expi293 expression medium with 100 μM biotin. RBDs in the culture supernatant were purified with TALON columns (Clontech).

The biotinylated RBDs were conjugated with streptavidin MagPlex beads (Luminex), washed and stored in PBS supplemented with 1% BSA, 0.05% tween 20, and 0.1% sodium azide (wash buffer). The beads were stirred with plasma sample diluted in PBS containing 1% BSA, 1% skim milk, 0.05% tween-20, and 0.1% sodium azide (assay diluent) in 96-well plate. The plasma samples were diluted to a uniform anti-RBD IgG titer prior to the experiment. The plates were incubated at 4°C overnight followed by washing with the wash buffer for three times. The beads were resuspended in assay diluent containing PE-conjugated goat anti-human IgG antibody (Southern Biotech) and incubated at room temperature for 1 h. After washing with the wash buffer for three times, the beads were resuspended in the wash buffer. The fluorescent intensity of PE was measured with Luminex xMap INTELLIFLEX system (Thermo Fisher Scientific). Plasma IgG antibodies binding each RBD were titrated to reference monoclonal antibodies, REGN10933 and COVA1-16. The titers were normalized to those against the unmutated ancestral RBD.

### Competition assay

Binding competition of reference Fab against plasma IgG was assessed with Luminex multiplex assay. RBD-conjugated beads were mixed with Fab in PBS-T containing 1% BSA and 1% skim milk (assay diluent) in 96-well plates, followed by incubation at 4 °C overnight. Aliquot plasma samples diluted in the assay diluent were added into the well. The plates were incubated at room temperature for 2 h, followed by wash with 1% BSA/PBS-T for three times. The beads were further incubated with PE-conjugated goat anti-human IgG antibody diluted in the assay diluent at room temperature for 1 h. After washing with 1%BSA/PBS-T for three times, the beads were suspended in 1% BSA/PBS-T, and fluorescent intensity was measured with Luminex xMap INTELLIFLEX system (Thermo Fisher Scientific). Fold changes of plasma IgG binding to RBD with epitope masking by each Fab over masking by control non-binding Fab which is specific to rabies virus G protein.

### Binding assay of monoclonal antibodies

Binding of monoclonal antibodies to spike proteins were measured with Luminex multiplex assay. WT and S2P spikes purchased from BioServUK (Cat.No. BSV-COV-PR-33) and Miltenyi Biotec (Cat.No. 130-127-683), respectively, were conjugated to beads by amine-coupling. Both spike proteins harbor mutations in a Furin cleavage site (PPAR to GSAS at residues 682-685). The beads were mixed with monoclonal antibodies in assay diluent in 96-well plates, followed by incubation at 4 °C overnight, and wash with 1% BSA/PBS-T for three times. The beads were further incubated with PE-conjugated goat anti-human IgG antibody diluted in the assay diluent at room temperature for 1 h. After washing with 1%BSA/PBS-T for three times, the beads were suspended in 1% BSA/PBS-T, and fluorescent intensity was measured with Luminex xMap INTELLIFLEX system (Thermo Fisher Scientific). Binding of the monoclonal IgG to each spike were normalized to reference C002 antibody.

### Molecular dynamics simulations

All molecular dynamics simulations were based on the previously published, fully glycosylated prefusion SARS-CoV-2 spike trimer model^60^, originally derived from PDB ID 6VSB^9^. In this published model, missing loops and unresolved regions had already been modeled and all experimentally resolved N-linked glycans were included, and mutations, including the proline substitutions at positions 986 and 987 and modifications at the furin cleavage site (682–685), had been reverted to the wild-type sequence. The prefusion-stabilized S2P construct was generated by reintroducing the K986P and V987P mutations, while maintaining identical glycosylation, protonation states, and overall system composition between the WT and S2P models. All systems were solvated using the CHARMM-GUI^61^, default protocol, and NaCl was added to neutralize the systems and achieve physiological ionic strength. The total number of atoms was 773,263 and 773,152 for 2P and WT simulation respectively, with box size around 200 Å.

Molecular dynamics simulations were performed using the GENESIS 2.1 molecular dynamics engine on the Fugaku supercomputer^62^. Simulation protocols followed exactly the same procedures used in our previous studies of SARS-CoV-2 spike dynamics, including our simulation of the D614G mutation^54,63^. After energy minimization and equilibration, production simulations were carried out for 500 ns under NVT ensemble. All simulations were initiated from an RBD-Up conformation, and identical force field parameters, integrator settings, and coupling schemes were applied to ensure direct comparability between the WT and 2 P systems.

Trajectory analyses were conducted using tools implemented in GENESIS 1.7 together with in-house scripts. RBD–SD1 hinge angles, RBD twist angles were defined by the center of mass of the Cα atoms in the RBD core (residues 335–466 and 491–526), the SD1 core (residues 324–329, 531–590), and the top residues of SD1 (residues 328, 329, 530, 531, 543, and 544). In the twist angle, one more point was added at the lower part of RBD (residues 335, 336, 361, 362, 524, and 525). The inter-domain distances were defined using centers of mass of selected residue groups for RBD core and residues 57 to 271 for NTD. Contact occupancies, salt bridges, and hydrogen bonds were quantified as the percentage of simulation frames in which the corresponding interaction criteria were satisfied using GetContacts program. Free-energy landscapes were constructed by projecting trajectory data onto selected angular coordinates and applying Boltzmann inversion. Structural visualization and representative conformations were generated using PyMOL, and probability distributions and free-energy plots were produced using gnuplot and Matplot, respectively.

## Supplementary information

Oishi-et-al_npjVaccines_SupplementaryFig

## Data Availability

The PDB and PSF files for the wild-type (WT) trimeric 1Up-Spike and mutant (2 P) complexes, prepared both with and without ions and explicit solvent, along with topology and force field files and a representative 620-frame trajectory covering the full simulation, have been deposited in the public repository Zenodo and are available at 10.5281/zenodo.19622297. The other datasets generated and/or analyzed in the present study are not publicly available as they form part of ongoing research but are available from YT on reasonable request.

## References

[1] Burton, D. R. Antiviral neutralizing antibodies: from in vitro to in vivo activity. *Nat. Rev. Immunol.* **23**, 720–734 (2023). 37069260 10.1038/s41577-023-00858-w PMC10108814

[2] Polack, F. P. et al. Safety and efficacy of the BNT162b2 mRNA Covid-19 vaccine. *N. Engl. J. Med.* **383**, 2603–2615 (2020). 33301246 10.1056/NEJMoa2034577 PMC7745181

[3] Baden, L. R. et al. Efficacy and safety of the mRNA-1273 SARS-CoV-2 vaccine. *N. Engl. J. Med.* **384**, 403–416 (2021). 33378609 10.1056/NEJMoa2035389 PMC7787219

[4] Gilbert, P. B. et al. Immune correlates analysis of the mRNA-1273 COVID-19 vaccine efficacy clinical trial. *Science* **375**, 43–50 (2022). 34812653 10.1126/science.abm3425 PMC9017870

[5] Earle, K. A. et al. Evidence for antibody as a protective correlate for COVID-19 vaccines. *Vaccine* **39**, 4423–4428 (2021). 34210573 10.1016/j.vaccine.2021.05.063 PMC8142841

[6] Vogel, A. B. et al. BNT162b vaccines protect rhesus macaques from SARS-CoV-2. *Nature* **592**, 283–289 (2021). 33524990 10.1038/s41586-021-03275-y

[7] Walsh, E. E. et al. Safety and immunogenicity of two RNA-based Covid-19 vaccine candidates. *N. Engl. J. Med.* **383**, 2439–2450 (2020). 33053279 10.1056/NEJMoa2027906 PMC7583697

[8] Jackson, L. A. et al. An mRNA vaccine against SARS-CoV-2 - preliminary report. *N. Engl. J. Med.* **383**, 1920–1931 (2020). 32663912 10.1056/NEJMoa2022483 PMC7377258

[9] Wrapp, D. et al. Cryo-EM structure of the 2019-nCoV spike in the prefusion conformation. *Science* **367**, 1260–1263 (2020). 32075877 10.1126/science.abb2507 PMC7164637

[10] Chen, B., Farzan, M. & Choe, H. SARS-CoV-2 spike protein: structure, viral entry and variants. *Nat. Rev. Microbiol.* **23**, 455–468 (2025). 40328900 10.1038/s41579-025-01185-8 PMC13137867

[11] Hoffmann, M. et al. SARS-CoV-2 cell entry depends on ACE2 and TMPRSS2 and is blocked by a clinically proven protease inhibitor. *Cell* **181**, 271–280.e8 (2020). 32142651 10.1016/j.cell.2020.02.052 PMC7102627

[12] Zhou, P. et al. A pneumonia outbreak associated with a new coronavirus of probable bat origin. *Nature* **579**, 270–273 (2020). 32015507 10.1038/s41586-020-2012-7 PMC7095418

[13] Walls, A. C. et al. Structure, function, and antigenicity of the SARS-CoV-2 spike glycoprotein. *Cell* **181**, 281–292.e6 (2020). 32155444 10.1016/j.cell.2020.02.058 PMC7102599

[14] Tong, P. et al. Memory B cell repertoire for recognition of evolving SARS-CoV-2 spike. *Cell* **184**, 4969–4980.e15 (2021). 34332650 10.1016/j.cell.2021.07.025 PMC8299219

[15] Turoňová, B. et al. In situ structural analysis of SARS-CoV-2 spike reveals flexibility mediated by three hinges. *Science* **370**, 203–208 (2020). 32817270 10.1126/science.abd5223 PMC7665311

[16] Barnes, C. O. et al. SARS-CoV-2 neutralizing antibody structures inform therapeutic strategies. *Nature* **588**, 682–687 (2020). 33045718 10.1038/s41586-020-2852-1 PMC8092461

[17] Chen, Y. et al. Broadly neutralizing antibodies to SARS-CoV-2 and other human coronaviruses. *Nat. Rev. Immunol.* **23**, 189–199 (2023). 36168054 10.1038/s41577-022-00784-3 PMC9514166

[18] Westendorf, K. et al. LY-CoV1404 (bebtelovimab) potently neutralizes SARS-CoV-2 variants. *Cell Rep.* **39**, 110812 (2022). 35568025 10.1016/j.celrep.2022.110812 PMC9035363

[19] Yuan, M. et al. A broad and potent neutralization epitope in SARS-related coronaviruses. *Proc. Natl. Acad. Sci. USA* **119**, e2205784119 (2022). 35767670 10.1073/pnas.2205784119 PMC9304036

[20] Yuan, M. et al. A highly conserved cryptic epitope in the receptor binding domains of SARS-CoV-2 and SARS-CoV. *Science* **368**, 630–633 (2020). 32245784 10.1126/science.abb7269 PMC7164391

[21] Piccoli, L. et al. Mapping neutralizing and immunodominant sites on the SARS-CoV-2 spike receptor-binding domain by structure-guided high-resolution serology. *Cell* **183**, 1024–1042.e21 (2020). 32991844 10.1016/j.cell.2020.09.037 PMC7494283

[22] Huang, K.-Y. A. et al. Structural basis for a conserved neutralization epitope on the receptor-binding domain of SARS-CoV-2. *Nat. Commun.* **14**, 311 (2023). 36658148 10.1038/s41467-023-35949-8 PMC9852238

[23] Xiong, X. et al. A thermostable, closed SARS-CoV-2 spike protein trimer. *Nat. Struct. Mol. Biol.* **27**, 934–941 (2020). 32737467 10.1038/s41594-020-0478-5 PMC7116388

[24] Garcia-Beltran, W. F. et al. COVID-19-neutralizing antibodies predict disease severity and survival. *Cell* **184**, 476–488.e11 (2021). 33412089 10.1016/j.cell.2020.12.015 PMC7837114

[25] Moriyama, S. et al. Temporal maturation of neutralizing antibodies in COVID-19 convalescent individuals improves potency and breadth to circulating SARS-CoV-2 variants. *Immunity* **54**, 1841–1852.e4 (2021). 34246326 10.1016/j.immuni.2021.06.015 PMC8249673

[26] Amanat, F. et al. SARS-CoV-2 mRNA vaccination induces functionally diverse antibodies to NTD, RBD, and S2. *Cell* **184**, 3936–3948.e10 (2021). 34192529 10.1016/j.cell.2021.06.005 PMC8185186

[27] Cho, A. et al. Anti-SARS-CoV-2 receptor-binding domain antibody evolution after mRNA vaccination. *Nature* **600**, 517–522 (2021). 34619745 10.1038/s41586-021-04060-7 PMC8674133

[28] Moriyama, S. et al. Structural delineation and computational design of SARS-CoV-2-neutralizing antibodies against Omicron subvariants. *Nat. Commun.* **14**, 4198 (2023). 37452031 10.1038/s41467-023-39890-8 PMC10349087

[29] Pinto, D. et al. Cross-neutralization of SARS-CoV-2 by a human monoclonal SARS-CoV antibody. *Nature* **583**, 290–295 (2020). 32422645 10.1038/s41586-020-2349-y

[30] Costello, S. M. et al. The SARS-CoV-2 spike reversibly samples an open-trimer conformation exposing novel epitopes. *Nat. Struct. Mol. Biol.* **29**, 229–238 (2022). 35236990 10.1038/s41594-022-00735-5 PMC9007726

[31] Hansen, J. et al. Studies in humanized mice and convalescent humans yield a SARS-CoV-2 antibody cocktail. *Science* **369**, 1010–1014 (2020). 32540901 10.1126/science.abd0827 PMC7299284

[32] Robbiani, D. F. et al. Convergent antibody responses to SARS-CoV-2 in convalescent individuals. *Nature* **584**, 437–442 (2020). 32555388 10.1038/s41586-020-2456-9 PMC7442695

[33] Liu, H. et al. Cross-neutralization of a SARS-CoV-2 antibody to a functionally conserved site is mediated by avidity. *Immunity* **53**, 1272–1280.e5 (2020). 33242394 10.1016/j.immuni.2020.10.023 PMC7687367

[34] Brouwer, P. J. M. et al. Potent neutralizing antibodies from COVID-19 patients define multiple targets of vulnerability. *Science* **369**, 643–650 (2020). 32540902 10.1126/science.abc5902 PMC7299281

[35] Zhang, Z. et al. Humoral and cellular immune memory to four COVID-19 vaccines. *Cell* **185**, 2434–2451.e17 (2022). 35764089 10.1016/j.cell.2022.05.022 PMC9135677

[36] Alameh, M.-G. et al. Lipid nanoparticles enhance the efficacy of mRNA and protein subunit vaccines by inducing robust T follicular helper cell and humoral responses. *Immunity* **54**, 2877–2892.e7 (2021). 34852217 10.1016/j.immuni.2021.11.001 PMC8566475

[37] Tahtinen, S. et al. IL-1 and IL-1ra are key regulators of the inflammatory response to RNA vaccines. *Nat. Immunol.* **23**, 532–542 (2022). 35332327 10.1038/s41590-022-01160-y

[38] Greaney, A. J. et al. Antibodies elicited by mRNA-1273 vaccination bind more broadly to the receptor binding domain than do those from SARS-CoV-2 infection. *Sci. Transl. Med.* **13**, eabi9915 (2021). 34103407 10.1126/scitranslmed.abi9915 PMC8369496

[39] Addetia, A. et al. Neutralization, effector function and immune imprinting of Omicron variants. *Nature* **621**, 592–601 (2023). 37648855 10.1038/s41586-023-06487-6 PMC10511321

[40] Yamin, R. et al. Fc-engineered antibody therapeutics with improved anti-SARS-CoV-2 efficacy. *Nature* **599**, 465–470 (2021). 34547765 10.1038/s41586-021-04017-w PMC9038156

[41] Case, J. B. et al. Resilience of S309 and AZD7442 monoclonal antibody treatments against infection by SARS-CoV-2 Omicron lineage strains. *Nat. Commun.* **13**, 3824 (2022). 35780162 10.1038/s41467-022-31615-7 PMC9250508

[42] Richardson, S. I. et al. SARS-CoV-2 beta and delta variants trigger Fc effector function with increased cross-reactivity. *Cell Rep. Med.* **3**, 100510 (2022). 35233544 10.1016/j.xcrm.2022.100510 PMC8761540

[43] Martin-Blondel, G. et al. Sotrovimab to prevent severe COVID-19 in high-risk patients infected with Omicron BA.2. *J. Infect.* **85**, e104–e108 (2022). 35803386 10.1016/j.jinf.2022.06.033 PMC9254651

[44] Cheng, M. M. et al. Real-world effectiveness of sotrovimab for the early treatment of COVID-19 during SARS-CoV-2 Delta and Omicron waves in the USA. *Infect. Dis. Ther.* **12**, 607–621 (2023). 36629998 10.1007/s40121-022-00755-0 PMC9832411

[45] Starr, T. N. et al. Deep mutational scanning of SARS-CoV-2 receptor binding domain reveals constraints on folding and ACE2 binding. *Cell* **182**, 1295–1310.e20 (2020). 32841599 10.1016/j.cell.2020.08.012 PMC7418704

[46] Kotaki, R. et al. SARS-CoV-2 Omicron-neutralizing memory B-cells are elicited by two doses of BNT162b2 mRNA vaccine. *Sci. Immunol.* **7,** eabn8590 (2022). 10.1126/sciimmunol.abn8590 PMC8939773 35113654

[47] Goel, R. R. et al. Efficient recall of Omicron-reactive B cell memory after a third dose of SARS-CoV-2 mRNA vaccine. *Cell* **185**, 1875–1887 (2022). 35523182 10.1016/j.cell.2022.04.009 PMC8989683

[48] Muecksch, F. et al. Increased memory B cell potency and breadth after a SARS-CoV-2 mRNA boost. *Nature* **607**, 128–134 (2022). 35447027 10.1038/s41586-022-04778-y PMC9259484

[49] Sokal, A. et al. Analysis of mRNA vaccination-elicited RBD-specific memory B cells reveals strong but incomplete immune escape of the SARS-CoV-2 Omicron variant. *Immunity* **55**, 1096–1104.e4 (2022). 35483354 10.1016/j.immuni.2022.04.002 PMC8986479

[50] Inoue, T. et al. Antibody feedback contributes to facilitating the development of Omicron-reactive memory B cells in SARS-CoV-2 mRNA vaccinees. *J. Exp. Med.* **220**, e20221786 (2023). 36512034 10.1084/jem.20221786 PMC9750191

[51] Schaefer-Babajew, D. et al. Antibody feedback regulates immune memory after SARS-CoV-2 mRNA vaccination. *Nature* **613**, 735–742 (2023). 36473496 10.1038/s41586-022-05609-w PMC9876794

[52] Wrobel, A. G. et al. Antibody-mediated disruption of the SARS-CoV-2 spike glycoprotein. *Nat. Commun.* **11**, 5337 (2020). 33087721 10.1038/s41467-020-19146-5 PMC7577971

[53] Zimmerman, M. I. et al. SARS-CoV-2 simulations go exascale to predict dramatic spike opening and cryptic pockets across the proteome. *Nat. Chem.* **13**, 651–659 (2021). 34031561 10.1038/s41557-021-00707-0 PMC8249329

[54] Dokainish, H. M. et al. The inherent flexibility of receptor binding domains in SARS-CoV-2 spike protein. *Elife*10.7554/eLife.75720 (2022). 10.7554/eLife.75720 PMC8963885 35323112

[55] Ye, G., Liu, B. & Li, F. Cryo-EM structure of a SARS-CoV-2 omicron spike protein ectodomain. *Nat. Commun.* **13**, 1214 (2022). 35241675 10.1038/s41467-022-28882-9 PMC8894419

[56] Gobeil, S. M.-C. et al. Structural diversity of the SARS-CoV-2 Omicron spike. *Mol. Cell* **82**, 2050–2068.e6 (2022). 35447081 10.1016/j.molcel.2022.03.028 PMC8947964

[57] Takano, T. et al. Distinct immune cell dynamics correlate with the immunogenicity and reactogenicity of SARS-CoV-2 mRNA vaccine. *Cell Rep. Med.* **3**, 100631 (2022). 35545084 10.1016/j.xcrm.2022.100631 PMC9023335

[58] Kotaki, R. et al. Repeated Omicron exposures redirect SARS-CoV-2-specific memory B cell evolution toward the latest variants. *Sci. Transl. Med.* **16**, eadp9927 (2024). 39167666 10.1126/scitranslmed.adp9927

[59] Takano, T. et al. Heterologous SARS-CoV-2 spike protein booster elicits durable and broad antibody responses against the receptor-binding domain. *Nat. Commun.* **14**, 1451 (2023). 36922492 10.1038/s41467-023-37128-1 PMC10016167

[60] Woo, H. et al. Developing a fully glycosylated full-length SARS-CoV-2 spike protein model in a viral membrane. *J. Phys. Chem. B* **124**, 7128–7137 (2020). 32559081 10.1021/acs.jpcb.0c04553 PMC7341691

[61] Jo, S., Kim, T., Iyer, V. G. & Im, W. CHARMM-GUI: a web-based graphical user interface for CHARMM. *J. Comput. Chem.* **29**, 1859–1865 (2008). 18351591 10.1002/jcc.20945

[62] Jung, J. et al. GENESIS 2.1: High-performance molecular dynamics software for enhanced sampling and free-energy calculations for atomistic, coarse-grained, and quantum mechanics/molecular mechanics models. *J. Phys. Chem. B* **128**, 6028–6048 (2024). 38876465 10.1021/acs.jpcb.4c02096 PMC11215777

[63] Dokainish, H. M. & Sugita, Y. Structural effects of spike protein D614G mutation in SARS-CoV-2. *Biophys. J.* **122**, 2910–2920 (2023). 36397671 10.1016/j.bpj.2022.11.025 PMC9671695

