# Supplementary material for: Prefusion-stabilized SARS-CoV-2 spike reshapes antigenic hierarchy and antibody targeting against conserved and occluded epitopes
Source: NPJ Vaccines. 2026 Apr 25;11:128. doi: 10.1038/s41541-026-01464-2 (PMC13324858; doi:10.1038/s41541-026-01464-2)
Supplement: Supplementary file 1 — Oishi-et-al_npjVaccines_SupplementaryFig [file 41541_2026_1464_MOESM1_ESM.pdf]

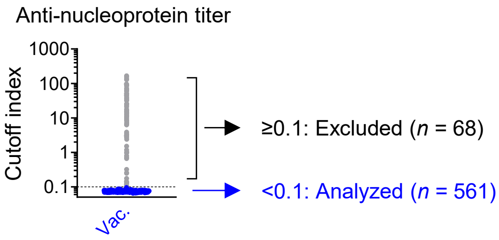

Fig. S1: Plasma antibody responses against SARS-CoV-2 nucleoprotein in the vaccinated participants.
Anti-nucleoprotein antibody titer were measured. Samples with values above 0.1 (indicated by a dotted line) were excluded from subsequent analyses as suspected to be infected by SARS-CoV-2.

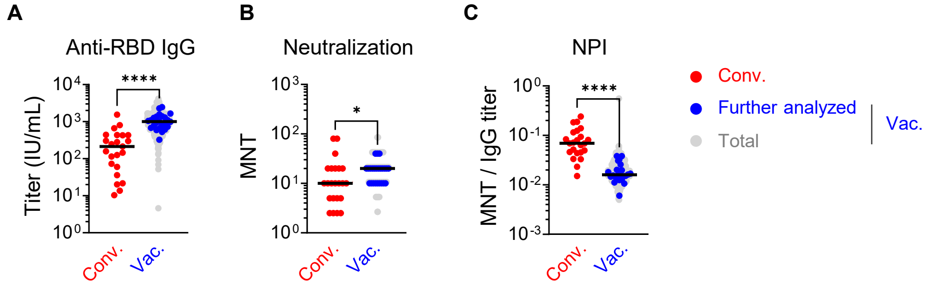

Fig. S2: Plasma antibody responses in the vaccinated participants further analyzed in detail.
Anti-RBD IgG titer (A), neutralization titer against authentic SARS-CoV-2 (B), and NPI calculated as the neutralization titer per the IgG titer (C) were compared between the convalescent and vaccinated plasma which were further analyzed in later experiments. Statistical analyses were performed with Mann-Whitney test (*$p < 0.05$, ****$p < 0.0001$) between the Conv. And Further analyzed Vac. groups. The dots represent data from each participant. The horizontal bars indicate median.

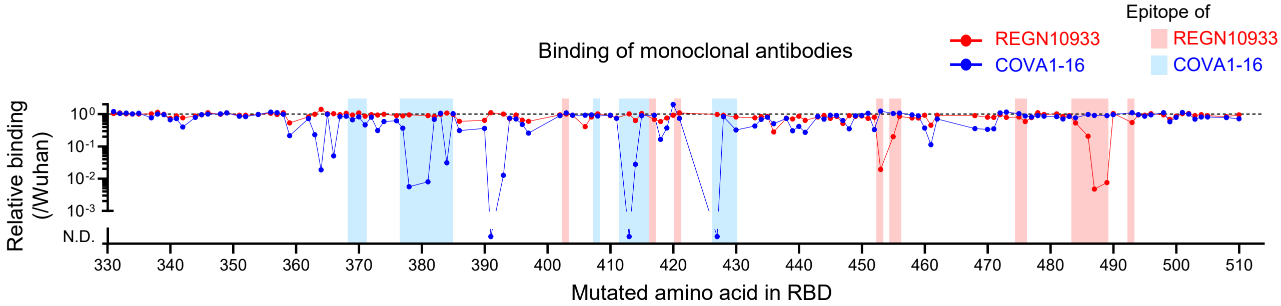

Fig. S3: Binding of monoclonal antibodies to a panel of RBD variants.
Relative binding to each single mutation normalized by binding to the ancestral RBD were analyzed for the monoclonal antibodies, REGN10933 (red) and COVA1-16 (blue). The amino acid residues reported to be epitope of each antibody are highlighted. N.D.indicates not detected.

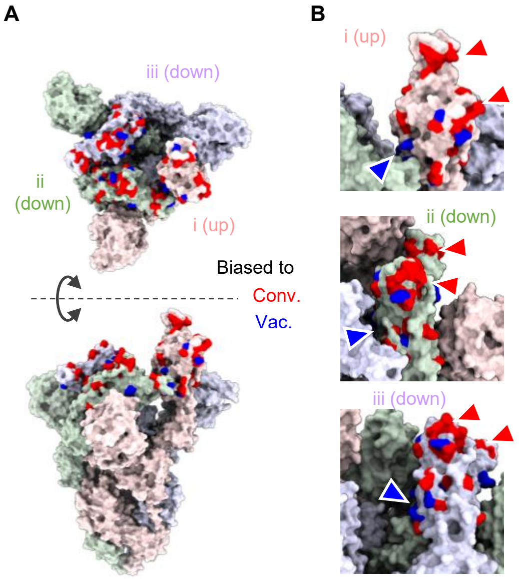

Fig. S4: The preferential epitopes in vaccinated and convalescent plasma on spike structure in conventional up- and down-RBDs.
The amino acid residues preferentially targeted by antibodies in convalescent (red) and vaccinated (blue) plasma indicated in Fig. 3G are highlighted in spike protein structure with 1 up-RBD (PDB: 7A94). The images were created with ChimeraX software.
A. Top and side views of whole image are shown.
B. Each of the three RBDs on the spike is focused. The clustered epitopes described in Fig. 3G are indicated by arrowheads.

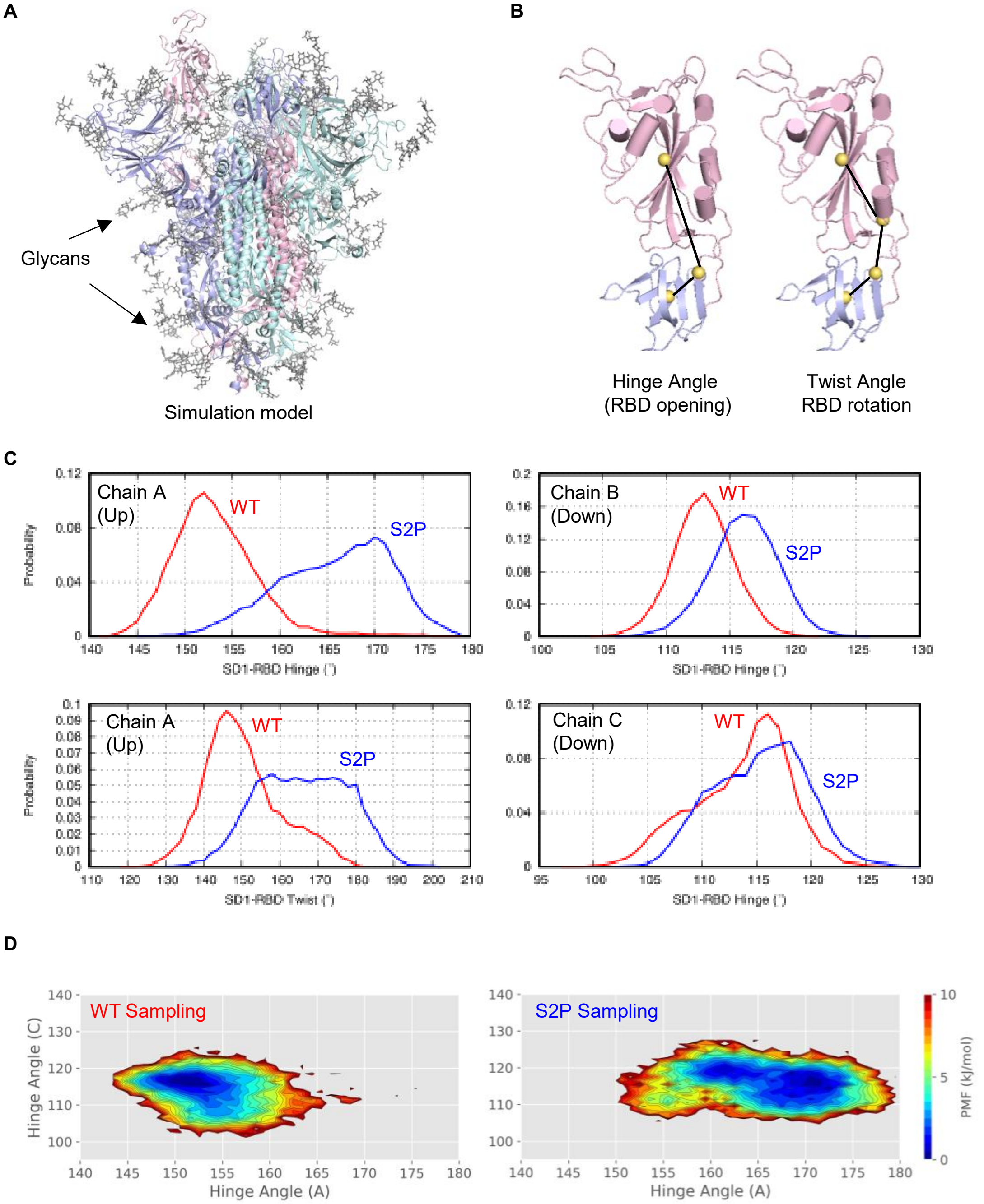

Fig S5: Definition of hinge and twist angles and ensemble-wide RBD conformational sampling across all protomers.
A. Cartoon representation of the simulation model including all modeled N-linked glycans (shown as sticks).
B. Schematic definition of the RBD hinge and twist angles relative to subdomain 1 (SD1). Centers of mass used to define the angles for the RBD and SD1 are shown as yellow spheres.
C. Probability distributions of the RBD–SD1 hinge angle for Chain A (Up) and Chains B and C (Down), together with the twist angle distribution for Chain A.
D. Two-dimensional free-energy landscape projected onto the hinge angles of Chain A (Up) and Chain C (Down), demonstrating that opening motions of the Up RBD occur largely independently of hinge fluctuations in the adjacent Down protomer.

A

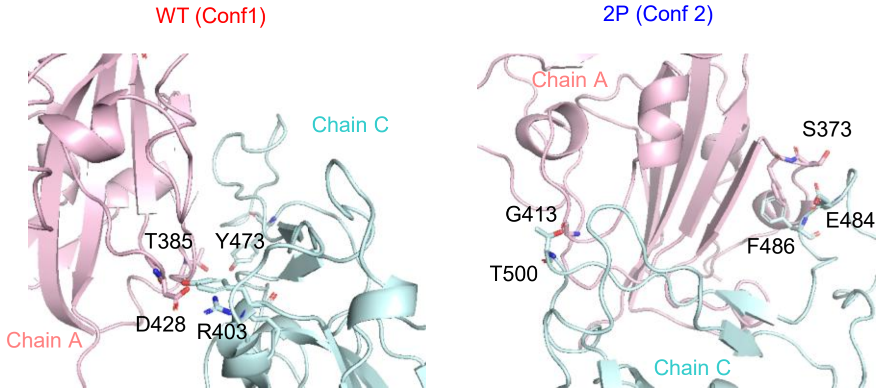

B

Chain A	Chain C	WT	S2P
ARG:403	ASP:428	79.1	0
THR:385	TYR:473	74.9	24.2
ASP:428	TYR:505	72.1	0.5
PHE:377	TYR:489	69.3	51.5
PHE:486	SER:371	40.0	62.5
PHE:374	PHE:486	26.8	78.3
LEU:368	PHE:486	3.2	88.0
SER:373	GLU:484	0.2	61.3
PHE:374	GLU:484	0	79.6
GLY:413	THR:500	0	79.1

C

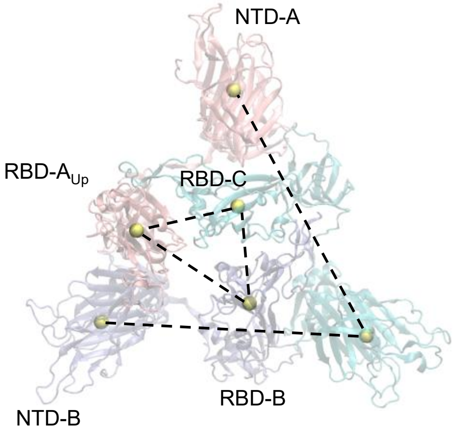

D

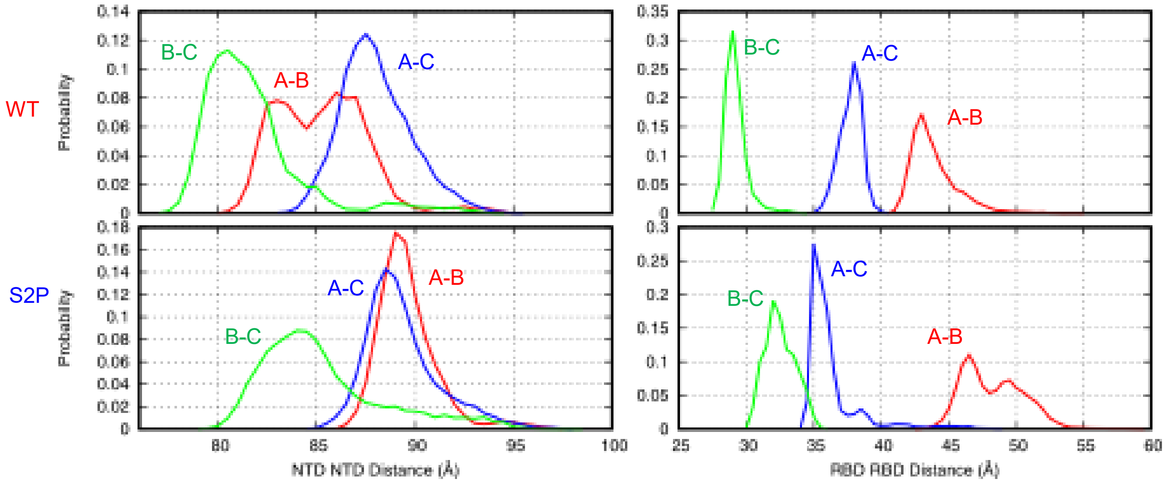

Fig S6: Inter-RBD interactions and S1 conformational heterogeneity in WT and S2P spike simulations.

A. Cartoon representation of interactions between RBD A (Up) and RBD C, with representative interfacial residues shown as sticks.

B. Occupancy (%) of RBD A–RBD C van der Waals contacts over the 500 ns simulations for WT and S2P, illustrating differences in contact patterns between WT and S2P simulations.

C. Cartoon representation of the S1 domain highlighting the relative arrangement of RBDs and N-terminal domains (NTDs). Centers of mass used to compute inter-domain distances are indicated.

D. Probability distributions of inter-NTD distances (left) and inter-RBD distances (right) for WT and S2P simulations, showing broader distance distributions in the S2P ensemble, consistent with increased conformational heterogeneity within S1.

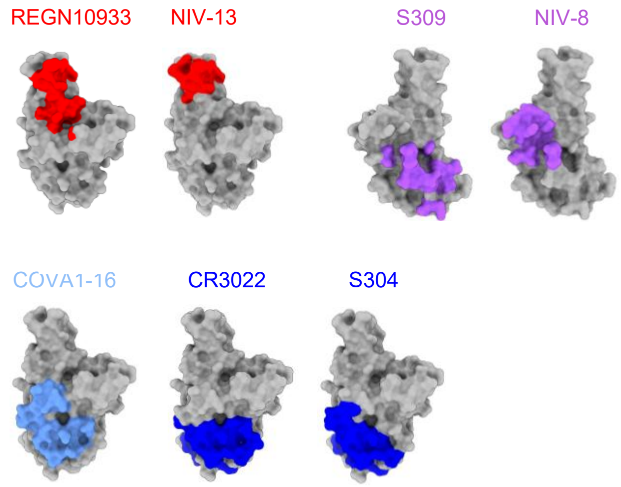

Fig. S7: The epitopes of the analyzed monoclonal antibodies.
Reported epitopes of the monoclonal antibodies analyzed in Fig. 4E, F are highlighted. The RBD structure (PDB: 7E5O) was analyzed with ChimeraX software.
